# Acute effects of a high fat and high carbohydrate meal with and without subsequent physical activity on cardiac autonomic modulation in people with type 2 diabetes

**DOI:** 10.1038/s41598-025-19506-5

**Published:** 2025-09-17

**Authors:** Janis Schierbauer, Paul Zimmermann, Lea Reichel, Sascha W. Hoffmann, Helmut K. Lackner, Othmar Moser

**Affiliations:** 1 Div. Exercise Physiology & Metabolism, University of Bayreuth (GER), Bayreuth, Germany; 2 Department of Cardiology, Klinikum Bamberg, Bamberg, Germany; 3 Interdisciplinary Center of Sportsmedicine Bamberg, Bamberg, Germany; 4 Div. Theory and Practice of Sports and Fields of Physical Activity, University of Bayreuth (GER), Bayreuth, Germany; 5 Department of Physiology, Medical University of Graz (AT), Graz, Austria; 6 Interdisciplinary Metabolic Medicine Research Group, Division of Endocrinology and Diabetology, Medical University of Graz (AT), Graz, Austria; 7 Exercise Physiology, Training & Training Therapy Research Group, Institute of Human Movement Science, Sport and Health, University of Graz (AT), Graz, Austria

**Keywords:** ACM, Holter, Electrocardiogram, glucose, Fructose, Palm fat, Treadmill walking, Endocrine system and metabolic diseases, Nutrition

## Abstract

The impact of different dietary interventions on autonomic cardiac modulation (ACM) in people with type 2 diabetes (T2D) is a remarkable, albeit scarcely studied topic. The aim of this secondary outcome analysis was to evaluate the effects of a high-carbohydrate (CHO) and high-fat meal (FAT) with and without subsequent physical activity (PA, CHO + PA and FAT + PA) on baseline electrocardiographic and heart rate variability (HRV) parameters in people with T2D. Eleven individuals with T2D (five females, 65.8 ± 5.9 years, body mass index: 29.5 ± 5.2 kg·m^−2^, HbA_1c_: 7.0 ± 0.8%) participated in this study. Electrocardiogram (ECG) and HRV parameters before and after the meal intake as well as during physical activity, a 30-minute self-paced treadmill walk, were analyzed. Baseline ECG parameters included resting heart rate (73 ± 8 beats per minute), PQ interval (160 ± 24 ms), QRS interval (92 ± 11 ms), QT (387 ± 17 ms) and QTc time interval (420 ± 20 ms) without clinically relevant dynamics. No negative impact of either dietary interventions or PA on HRV parameters, i.e. standard deviation of the NN interval (SDNN, (F [1.9] = 0.28), root mean square of successive differences (RMSSD, (F [1.9] = 0.09) and ln LF/HF (low frequency/high frequency) (F [1.9] = 0.00) could be revealed displaying preserved ACM. In conclusion, our secondary outcome analysis displayed preserved ACM in T2D independently of dietary intervention or additional PA. Finally, our research might strengthen the scientific data on dietary intervention in T2D and demonstrate future preventive options in the context of T2D and nutrition.

## Introduction

Type 2 diabetes mellitus (T2D) represents a metabolic imbalance of insulin needs and sensitivity caused by multiple etiological factors resulting in clinical features, such as hyperglycemia, insulin resistance and insufficient insulin secretion^1^. The incidences and prevalences of T2D have continuously increased over the last decades and are currently affecting one in ten individuals aged 20 to 79 years worldwide^2^. It is estimated that nearly 785 million people worldwide will be diagnosed with T2D by the year 2025^2,3^.

Type 2 diabetes mellitus is considered to display an important cardiovascular risk factor, as previous research revealed an increased risk for individuals with diabetes for major adverse cardiovascular events (MACE), such as acute myocardial infarction or ischemic stroke^4,5^. Next to various T2D related clinical abnormalities and dysregulation, individuals suffering from T2D may develop an autonomic nervous system (ANS) alteration in general and in detail a dysfunction of the cardiovascular system, i.e. cardiovascular autonomic neuropathy (CAN), during their clinical course in life^4,5^. Previous research revealed the prevalence for subclinical CAN in people with T2D up to 34.3% based on abnormal findings in heart rate variability (HRV) tests^6^. CAN is associated with impaired autonomic regulation and feedback mainly affected by lesions in the peripheral sympathetic and parasympathetic autonomic fibers^7^. Diabetic autonomic neuropathy (DAN) in general, characterized by alterations and imbalance within the parasympathetic nervous system, i.e. vagal system, and the sympathetic nervous system, is a serious and common short or long-term complication in diabetes^4,8^. The prevalence of DAN is estimated to affect up to 20% of asymptomatic people with diabetes, partly as a coexisting disorder with other peripheral neuropathies as well as an isolated dysregulation^8^. Major clinical manifestations of DAN are covering autonomic failure in the context of orthostatic hypotension, reduced hypoglycemic awareness, gastroparesis, erectile dysfunction as well as exercise intolerance, inappropriate tachycardia tendency and silent myocardial ischemia^4,8^. Several epidemiological studies have proven in this context a five-time higher mortality for individuals with CAN^8^. CAN is the most prominent and professionally researched autonomic disorder in the context of diabetes because of its life-threatening consequences due to complications and the established diagnostic testing options, such as HRV measurements and analyses.

In this context, previous research has demonstrated only modest short-term benefits of exercise training - both resistance and aerobic - for the prevention and treatment of diabetic autonomic neuropathy (DAN)^9,10^. For instance, oxidative stress, a well-established key pathophysiological mechanism in the development of DAN^11^, may be positively modulated by several emerging therapeutic strategies. Nevertheless, exercise may still play a meaningful role in this context, as the concept of “exercise as medicine” has been previously highlighted in the management of metabolic diseases, including type 2 diabetes (T2D)^12^. Therefore, exercise and physical activity, when tailored to the individual’s optimal type and dosage, contribute to improved management of T2D-related cardiovascular risk factors and overall prognosis. However, to date, only a few well-controlled studies have comprehensively evaluated the metabolic effects of high-carbohydrate versus high-fat meals in individuals with T2D. Evidence suggests that a diet high in unsaturated fats may improve glycemic control, support blood pressure regulation, and reduce the need for diabetes medications^13^. For instance, Beltrame et al. reported reduced parasympathetic modulation at rest and delayed heart rate recovery after exercise in subjects with T2D, findings that may be linked to increased cardiovascular morbidity and mortality^14^. Their findings also indicated impaired cardiac autonomic regulation, which was associated with elevated serum glucose levels.

Therefore, and due to the lack of broad scientific evidence in this field, the primary objective of this secondary outcome analysis was to investigate the acute effects of a high-carbohydrate (CHO) and high-fat (FAT) meal with and without subsequent physical activity (PA) on baseline HRV and electrocardiographic (ECG) parameters in people with T2D.

## Materials and methods

This single-centre, randomized, controlled crossover trial represents a secondary outcome analysis and investigates the effects of a CHO and FAT meal with and without subsequent PA on baseline ECG and HRV parameters in people with T2D. The study was conducted at the Div. Exercise Physiology & Metabolism, Bayreuth Centre of Sport Science, University of Bayreuth, Germany.

The study protocol was approved by the local ethics committee of the University of Bayreuth, Germany (23 − 011, 06/04/2023) and registered in the German Clinical Trials Register (DRKS-ID: DRKS00032569, 27/09/2023, https://www.drks.de/search/de/trial/DRKS00032569). The primary outcome of the registered study was to investigate the acute effects of a high-fat and high-carbohydrate meal with and without subsequent PA on the inflammatory immune response, i.e., interleukin-6 in adult individuals with T2D. In this secondary outcome analysis, however, the same participants (and thus number of participants) were analyzed as in the main study. The study was planned and carried out in accordance with the principles of Good Clinical Practice and the Declaration of Helsinki^15^. Potential participants were informed about the study protocol and provided written informed consent before any trial-related examinations were conducted.

### Inclusion and exclusion criteria

Inclusion criteria included male or female participants with a clinically diagnosed T2D aged between 18 and 85 years and a body mass index ≤ 39.9 kg·m^−2^. Additionally, glycated hemoglobin A_1c_ (HbA_1c_) had to be within the range of 6.5 and 9.5% (47.5–80.3 mmol·mol^−1^).

Exclusion criteria included the following: Simultaneous enrolment in any other study, known or suspected hypersensitivity to trial products or related products, receipt of any investigational medicinal product within 1 week prior to screening in this trial, suffering from or history of a life-threatening disease (i.e. cancer judged not to be in full remission except basal cell skin cancer or squamous cell skin cancer), or clinically severe diseases that directly influence the study results (this did not prohibit the participation of people taking medications that influence the metabolism (e.g. statin) or cardio-respiratory system (e.g. asthma spray) as long as the therapy is stable and not adapted throughout the run of the trial), resting heart rate < 35 beats per minute and blood pressure outside the range of 90–150 mm Hg for systolic or 50–95 mm Hg for diastolic after resting for five minutes in a supine position at the screening visit (excluding white-coat hypertension; therefore, if a repeated measurement on a second screening visit shows values within the range, the participant can be included in the trial), significant abnormal ECG at screening, as judged by the medical investigator, any chronic (metabolic) disorder other than T2D or severe disease which, in the opinion of the investigator, might jeopardize the participant’s safety or compliance with the protocol, a history of multiple and/or severe allergies to drugs or foods or a history of severe anaphylactic reaction, a history of alcoholism or drug abuse, participants with mental incapacity or language barriers including adequate understanding or cooperation or who, in the opinion of their general practitioner or the investigator, should not participate in the trial, any condition that would interfere with trial participation or evaluation of results, females of childbearing potential who intend to become pregnant or are pregnant, or are not using adequate contraceptive methods (adequate contraceptive measures include sterilization, hormonal intrauterine devices, oral contraceptives, sexual abstinence or vasectomized partner, this also includes females who are breast-feeding).

### Assessment of eligibility

Eligibility criteria were assessed by the same investigator at the screening visit.

### Study design and setting

This is a randomized, controlled crossover trial in a laboratory setting including one screening visit and four trial related visits (V1-V4). All visits took place at the laboratories of the research facility (Division Exercise Physiology & Metabolism, Bayreuth Centre of Sport Science, University of Bayreuth) and were conducted in conformity with the local COVID-19 guidelines, which included the screening of any COVID-19 related symptoms prior to each of the trial related visits. If, before any visit, a participant felt uncomfortable or was deemed sick by the study team, the participant was sent home and the visit rescheduled.

### Screening visit

At the screening visit, participants were informed about all study-related procedures, given instructions for the study, and provided informed consent. Afterwards they were examined for their general health status via a physical examination including the automatic measurement of blood pressure (BU 510 Blood Pressure Monitor, Medisana GmbH, Neuss, Germany) after resting in a supine position for five minutes and afterwards a 12-lead electrocardiogram (ECG) recording (Amedtec ECGpro^®^, Cardiopart 12, Straessle & Co. Medizintechnik GmbH, Albstadt, Germany) in lying position. The resting 12-lead ECG was analyzed with regard to baseline electrocardiographic parameters, including heart rate and ECG time interval measurements, i.e., PQ interval, QRS interval, QT and QTc time interval dynamics as reported previously^16^. The computerized measurements of baseline ECG parameters were performed automatically using the ECG assessment software. Afterwards, all study participants were critically evaluated for the following suspected ECG abnormalities: potential clinically relevant sinus bradycardia (predefined as resting HR < 60 beats per minute), physiological atrioventricular blocks (AVB, defined as the first and second degree AVB’s), abnormal right and left axis aberration (defined as more positive than 110° or more negative than 0°), pathological QRS interval prolongation estimated as QRS lengthening > 120 ms or criteria of preexcitation syndromes, as well as early repolarization (ER) patterns. No participant had to be excluded due to abnormal baseline ECG parameters.

A venous blood sample was drawn from the antecubital vein for a large blood count (6 mL ethylenediaminetetraacetic acid). Body composition was analyzed in duplicate using a bioelectrical impedance analysis (InBody 720, InBody, Seoul, South Korea). Afterwards, participants were asked about the types of intensity of physical activity and sitting time as part of their daily lives using the International Physical Activity Questionnaire (IPAQ-SF)^17^. Participants were also asked to maintain their normal physical activity during their study participation. Before leaving the study center, participants were assigned to ascending numbers and then allocated to the order in which the trial visits were conducted following a cross-over, randomized fashion using the software Research Randomizer^®^ (1:1:1:1)^18^. Random allocation sequence was generated by the primary investigator.

### Trial-related visits (V1-4)

At the start of each trial visit, participants were screened for the testing day´s inclusion and exclusion criteria. For instance, participants had to be in an overnight-fasted state and refrain from alcohol consumption and any physical exercise on the day prior to the trial visit. A Holter-ECG (Bittium Faros 180, Bittium Corp., Oulu, Finland) was placed on the participants chest according to the manufacture’s guidelines. Participants then received one of two meals: A high-carbohydrate (CHO) drink containing 1 g·kg − 1 of CHO consisting of 0.5 g·kg^−1^ glucose (D(+)-Glucose x H2O 99%, Grüssing GmbH, Filsum, Germany) and 0.5 g·kg^−1^ fructose (D(−)-Fructose 99%, Grüssing GmbH, Filsum, Germany) dissolved in 300 mL of water or a high-fat meal containing 1 g·kg^−1^ of palm oil (Stübener Kräutergarten, Dornbirn, Austria) mixed with high-fat Greek style yoghurt (3.5%, REWE Markt GmbH, Cologne, Germany). The amounts of carbohydrates and fats consumed was standardized and given based on the participants body mass, which was determined at the start of each trial visit using a commercially available digital scale (Seca Clara 803, Seca, Hamburg, Germany). In the case of the high-fat meal, however, a minimum amount of 80 g palm fat was specified. Both meals had to be consumed within 5 min, and a timer and the Holter-ECG were started when the meal was finished.

**Fig. 1:**
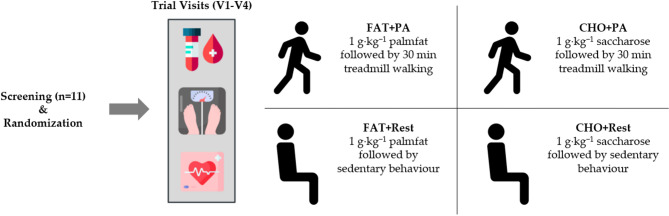
Study Flow Chart (FAT = high fat palm oil meal, CHO = high carbohydrate meal, PA = 30-minute treadmill walking, Rest = resting in a seated position).

After the meal consumption, participants initially remained in a seated position for a minimum of 30 min and then followed one of four conditions in a randomized order: They remained seated until 3-hours post-meal consumption (FAT + Rest and CHO + Rest) or they performed a 30-minute treadmill walking protocol (FAT + PA and CHO + PA). Participants were walking on a standardized incline of 1% and chose a self-selected velocity that was replicated during the other trial visit. After completion of the 30-minute treadmill walking, participants once again returned to a seated position and remained there until the end of the visit, which was 180 min post-meal consumption. The study flow chart can be found in Figure. 1.

### ECG assessment

The recorded 12-lead ECGs were analyzed with regard to baseline electrocardiographic parameters, including heart rate (HR) and ECG time interval measurements, i.e., PQ interval duration (ms), QRS interval pattern (ms) and QTc time interval dynamics (ms)^19^. The computerized measurements of baseline ECG parameters were performed digitally using the Amedtec–ECG assessment software (CardioPart 12, Amedtec, Aue-Bad Schlema, Germany). All study participants were critically evaluated for the following suspected ECG abnormalities: potential clinical relevant sinus bradycardia (predefined as resting HR < 60 bpm in a supine position for five minutes); physiological atrioventricular blocks (AVB), defined as the first and second (Mobitz I) degree AVBs; abnormal right and left axis aberration (defined as more positive than 110 or more negative than 0); pathological QRS interval prolongation (estimated as QRS lengthening > 120 ms) or criteria of preexcitation syndromes, as well as early repolarization (ER) patterns. Upon this critical evaluation, no participant had to be eliminated due to abnormal baseline ECG parameters.

### HRV assessment

The long-term Holter-ECG recording (Bittium Faros 180, Bittium Corp., Oulu, Finland) used in this trial utilized one channel with a 250-Hz sampling frequency^19^. During each of the four trial visits, the following ECG data measurements were evaluated to assess the sympathicovagal balance based on HRV analyses: The standard deviation of R-R intervals (SDNN), the square root of the mean standard difference of successive R-R intervals (RMSSD) and a logarithmic analysis referring to the ratio low frequency/high frequency, ln (LF/HF). A power spectral analysis regarding the frequency domain assessment was performed using Fast-Fourier Transformation in Cardiscope (Hasiba Medical GmbH, Graz, Austria). The balance of the autonomic nervous system was displayed by the HRV data evaluation of RMSSD and the ratio of low frequency/high frequency (ln, LF/HF).

For statistical analyses, three HRV phases after PA were each calculated for two-minute intervals, step size 30 s. intervals, receiving 60 epochs in each of the three predefined segments of 30 min. The average value for each 30-minute post-PA phase is calculated from these 60 segments and is shown in Table 3. HRV data were calculated starting with at 75 min post-meal consumption (or 15 min post-PA in the CHO + Ex and FAT + Ex trial arms). Therefore, the first period (I) included data from 75-minutes until 105-minutes post-meal consumption, the second period (II) from 105- to 135-minutes post-meal consumption and the third (III) from 135- to 165-minutes post-meal consumption. These periods are displayed in Table 3. This was done to validate the HRV calculations requiring quasi-stationary conditions. One example here is the dynamics of the heart rate decline, which would otherwise create incorrect ln(LF/HF) values. The HRV assessment and data acquisition were conducted based on the current Task Force guidelines of the European Society of Cardiology (ESC) and the recommendations of the North American Society of Pacing and Electrophysiology (NASPE)^20,21^.

### Power analysis

Sample size estimation was performed using G-Power (3.1.9.7) based on a similar project^22^ for the primary outcome of the study, which was the effect of high-fat/-carbohydrate meals (and PA) on the acute inflammatory response, i.e., interleukin 6 (IL-6) concentrations, in individuals with T2D. It resulted in an effect size of d = 0.975 (IL-6 levels in group 1: 4.1 ± 0.7 pg·mL^−1^, group 2: 8.3 ± 1.2 pg·mL^−1^). The level of significance was set at 0.05 and the power (1 − β error) at 0.80. This resulted in an a priori calculated sample size of six participants per group (since this was a crossover study the total number of participants required is also six). The aim for the main study, however, was to recruit a total of ten participants.

### Statistical analyses

All acquired data were processed in SPSS (IBM SPSS Statistics 28, IBM, New York, NY, USA) and assessed for normal distribution by the Shapiro–Wilk normality test. Descriptive statistics are presented according to their distribution as mean ± standard deviation, minima, and maxima and 95% confidence intervals (95% CI).

Data were analyzed via a repeated measure one-way analysis of variance (RM-ANOVA) for interaction differences. The interaction differences due to variable factors, such as influence of CHO or FAT ingestion, condition of PA or combination of both factors were taken into consideration by two-way ANOVA testing.

Adjusted post-hoc tests were performed via the Tukey test to explore the differences between the data means of the respective trial arms. Statistical significance was accepted at *p* < 0.05 (two-tailed).

## Results

### Participant characteristics

Twelve individuals with T2D were recruited between 06/2023 and 08/2023 with one participant leaving the study prematurely due to impaired cardiorespiratory fitness during the treadmill walking protocol. Hence, a total of eleven participants (five females) were included for statistical analyses with none of them being diagnosed with DAN at the time of their study participation.

(Diabetes-specific) Medications included, among others, GLP-1 receptor agonist (Dulaglutide, *n* = 1) Metformin (*n* = 7), Statins (*n* = 5), ACE inhibitors (*n* = 3), Basal insulins (*n* = 3).

Physical activity measurements assessed via the IPAQ-SF at the screening visit were 1375 ± 1772 MET-mins∙week^−1^ for walking, 1549 ± 1553 MET-mins∙week^−1^ for moderate, 1549 ± 1511 MET-mins∙week^−1^ for vigorous and 4473 ± 3134 MET-mins∙week^−1^ for total levels, respectively. Participant characteristics including body composition and diabetes-specific parameters are displayed in Table 1.

**Table 1:** Participant characteristics including body composition, and diabetes-specific parameters.

	Mean ± SD	Min - max	95% CI
Age (ys)	65.8 ± 5.9	55–76	61.7–70.0
Body mass (kg)	85.0 ± 20.5	54–118	70.6–99.4
Height (cm)	169 ± 9	151–180	162–176
Body Mass Index (kg·m^−2^)	29.5 ± 5.2	22.4–37.9	25.8–33.2
Intracellular water (L)	24.1 ± 5.6	15.2–34.9	20.2–28.1
Extracellular water (L)	15.0 ± 3.2	9.8–21.2	12.8–17.3
Fat mass (kg)	31.5 ± 12.4	13.8–52.3	22.8–40.2
Fat mass (%)	36.4 ± 8.4	19.6–54.5	30.5–42.3
Skeletal muscle mass (kg)	29.4 ± 7.3	17.9–43.5	24.3–34.6
Visceral fat area (cm^2^)	127 ± 45	19.6–54.5	95.0–159.0
HbA_1c_ (%)	7.0 ± 0.8	6.1–8.6	6.4–7.5
HbA_1c_ (mmol·mol^−1^)	52.7 ± 8.7	43.2–70.5	46.6–58.8
Diabetes duration (years)	16.1 ± 16.6	2–58	3.6–28.6

SD = standard deviation, Min = Minimum, Max = Maximum, CI = confidence interval, HbA_1c_ = glycated hemoglobin A_1c_.

### Meal intake and treadmill walking

Since body mass was recorded at each study visit and served as the basis for calculating carbohydrate and fat quantities, participants consumed varying amounts of both carbohydrates and fats during each trial arm: A total of 92.3 ± 14.0 g (CHO), 92.2 ± 14.1 g (CHO + PA), 92.2 ± 14.1 g (FAT) and 92.3 ± 14.3 g (FAT + PA) were consumed, respectively. The total caloric content of the meals was 378.2 ± 57.5 kcal (CHO), 378.2 ± 57.6 kcal (CHO + PA), 1029.8 ± 127.1 kcal (FAT) and 1031.0 ± 129.0 kcal (FAT + PA), respectively. For the fat trial arms (FAT and FAT + PA), these numbers already include an additional 200 kcal coming from the 300 g Greek style yoghurt. Mean self-selected velocity was 3.5 ± 0.5 km·h^−1^, ranging from 3.0 to 4.5 km·h^−1^.

### Baseline ECG

Baseline ECG parameters from the screening visit including HR, PQ, and QRS duration as well as QT and QTc intervals were normally distributed and are shown in Table 2. No clinically relevant pathologies and no significant severe abnormal ECG findings were detected, including atrioventricular blockings, QRS widening or complete branch blocking, any hints for preexcitation syndrome patterns or ER abnormalities.

**Table 2:** Baseline ECG parameters obtained during the screening visit presented as mean ± standard deviation, minimum and maximum as well as 95%-confidence interval. Values were recorded after resting in a supine position for five minutes.

	Mean ± SD	Min - max	95% Confidence interval
HR (1·min^−1^)	73 ± 8	63–92	66–79
PQ interval (ms)	160 ± 24	134–212	143–178
QRS interval (ms)	92 ± 11	74–108	85–100
QT interval (ms)	387 ± 17	356–408	375–399
QTc interval (ms)	420 ± 20	386–455	406–435

### Autonomic cardiac modulation (ACM)

ACM results in comparison of trial arms can be found in Table 3. F-statistics revealed a statistically significant effect of PA on HR (*p* = 0.016). Further HRV data analyses demonstrated no statistically significant differences for SDNN, RMSSD and the ratio of low frequency/high frequency, i.e., ln (LF/HF) for the variable effects of “CHO/FAT”, “PA” or “CHO/FAT x PA” by F-Statistic analyses (see footer Table 3). By detailed statistical consideration, no relevant differences in RMSSD assessment representing the parasympathetic part of the ANS, as well as in the ln (LF/HF) data analyses symbolizing sympathicovagal balance could be elucidated.

**Table 3:** Autonomic cardiac modulation (ACM) presented as mean ± standard deviation in comparison of trial arms including F-statistics. The column *‘time’* represents the three 30-minute time intervals for which the mean ACM values for each trial arm were calculated.

	Time	CHO	FAT	CHO + PA	FAT + PA	F-Statistics*	*P*-Value
HR (·min^−1^)	I	72.5 ± 9.9	69.9 ± 9.3	78.2 ± 10.7	76.2 ± 8.1	CHO/FAT: F(1.9) = 1.64	0.233
	II	70.5 ± 8.7	70.6 ± 8.7	75.8 ± 10.1	74.2 ± 9.5	PA: F(1.9) = 8.84	0.016
	III	68.8 ± 9.6	69.5 ± 9.9	73.4 ± 9.8	71.2 ± 8.1	CHO/FAT x PA: F(1.9) = 0.88	0.372
SDNN (ms)	I	43.3 ± 22.2	42.8 ± 25.3	38.3 ± 21.2	42.1 ± 26.9	CHO/FAT: F(1.9) = 0.03	0.864
	II	44.8 ± 21.5	44.5 ± 27.5	45.5 ± 24.3	44.4 ± 22.7	PA: F(1.9) = 0.30	0.595
	III	48.4 ± 24.0	48.0 ± 24.5	45.6 ± 22.2	46.2 ± 25.5	CHO/FAT x PA: F(1.9) = 0.28	0.611
RMSSD (ms)	I	23.2 ± 13.2	25.0 ± 17.5	24.2 ± 17.7	26.2 ± 29.2	CHO/FAT: F(1.9) = 0.23	0.643
	II	22.0 ± 12.1	25.4 ± 17.7	26.2 ± 17.8	24.3 ± 16.5	PA: F(1.9) = 0.00	0.980
	III	29.9 ± 14.9	26.2 ± 19.0	22.7 ± 13.3	24.9 ± 20.2	CHO/FAT x PA: F(1.9) = 0.09	0.771
ln (HF) (·ms^−2^)	I	4.35 ± 1.08	4.51 ± 1.19	4.10 ± 1.33	4.17 ± 1.44	CHO/FAT: F(1,9) = 0.70	0.423
	II	4.31 ± 0.97	4.50 ± 1.16	4.19 ± 1.30	4.30 ± 1.22	PA: F(1,9) = 0.86	0.378
	III	4.60 ± 0.95	4.54 ± 1.20	4.21 ± 1.13	4.47 ± 1.19	CHO/FAT x PA: F(1,9) = 0.05	0.832
ln (LF/HF)	I	1.65 ± 0.73	1.57 ± 0.73	1.59 ± 0.58	1.66 ± 0.58	CHO/FAT: F(1.9) = 0.20	0.666
	II	1.80 ± 0.66	1.61 ± 0.68	1.79 ± 0.47	1.79 ± 0.50	PA: F(1.9) = 0.11	0.745
	III	1.62 ± 0.59	1.72 ± 0.80	1.86 ± 0.43	1.65 ± 0.33	CHO/FAT x PA: F(1.9) = 0.00	0.990

SDNN = standard deviation of normal-to-normal beat, RMSSD = root mean square of successive differences, ln = logarithm, LF = low frequency, HF = high frequency.

In column *time*, I = first period displaying ACM data between 75- and 105-minutes post-meal consumption, II = between 105- and 135-minutes post-meal consumption, III = between 135- and 165-minutes post-meal consumption. HRV data was calculated starting 75 min after meal consumption (= 15 min post-PA) to validate ACM calculations requiring quasi-stationary conditions.

*CHO/FAT = main effect comes from trial arms without PA, PA = main effect comes from trial arms with physical activity, CHO/FAT x PA = main effect comes from both conditions.

## Discussion

The aim of this secondary outcome analysis of a randomized, controlled crossover trial in a laboratory setting was to investigate the acute effects of a high-carbohydrate and high-fat meal with and without subsequent PA on ECG and HRV parameters in people with T2D. Our main findings revealed no clinically relevant changes in HRV and ECG data after the intake of either a high-carbohydrate or a high-fat meal with post-prandial PA eliciting no favorable alterations in cardiac outcomes.

Previous research has shown generally lower sympathetic and higher parasympathetic cardiac modulation and subsequent HRV alterations in healthy young adults when glucose concentrations were acutely increased within the physiological range^23^. HRV analysis in people with T2D, however, has demonstrated controversial results. T2D is generally associated with an overall decrease in HRV, and both sympathetic and parasympathetic activity have been shown to be impaired by altered glucose metabolism, all possibly contributing to serious diabetic complications, i.e., DAN^24^. Therefore, the early detection of DAN and potential preservation of autonomic nervous system or delay of progression are of significant clinical importance^16^. Next to medicinal and/or therapeutical approaches, e.g., α- or β-blocker therapy and parasympathetic agonists, dietary interventions play an integral role in the future to maintain glycemic control, reduce chronic inflammation and improve long-term preservation of autonomic nervous system in people with T2D^25^.

The significant relationship between HRV and altered glucose metabolism might explain the altered parasympathetic and sympathetic activity, whereby the relationship between HbA1c levels and HRV has been proven in previous research^24^. Higher HbA1c levels were associated with shorter RR intervals and subsequent higher risk of ventricular arrhythmias, higher LF/HF ratio, and higher levels of SDNN^24,26,27^. Despite these established scientific data, the question arises as to the extent to which a modification of dietary habits and potential sub-sequent reduction of inflammation in T2D might positively influence the progression of DAN. The current scientific data on this topic is currently scarce. Previous research on differential effects of high-carbohydrate and high-fat meals on the sympathetic nervous system activity in lean and obese women without diabetes revealed no significant differences for fat ingestion and greater sympathetic response for carbohydrates in lean compared to obese women^28^. However, in obese people with T2D, energy restriction over 8 weeks resulted in improved cardiac vagal function and improved energy balance based on improved oxidative glucose utilization^29^.

Additionally, our findings emphasize preserved ACM due to variable dietary interventions in T2D and could not reveal imbalances of the sympathicovagal system based on HRV analyses which might be associated with an increased risk for arrhythmogenic substrates or future MACE. Therefore, the administrated high-carbohydrate and high-fat meal intervention on T2D subjects did not reveal any negative impact on ACM during our short-term laboratory evaluation and might open paths to future innovative dietary intervention strategies in T2D, as adapted for low-carbohydrate nutrition strategy.

In terms of the positive effect of PA on HRV parameters, it is likely that both duration and intensity of PA are the most potent factors to induce beneficial changes in ACM. While no data are available on ACM modulation as a result of post-prandial PA in people with T2D, previous research has demonstrated acute beneficial effects on post-prandial hyperglycemia, at least when undertaken as soon as possible after the meal intake^30^. The participants in this study started exercising exactly 30 min post-meal intake. Since there are no comparable studies, it can only be speculated whether this might have been too late to elicit changes in ACM. However, it should be emphasized that we explicitly used a standardized meal with food amounts based on the participants’ body mass. We recommend this approach for future studies, e.g., to systematically analyze and compare the influence of factors such as caloric content, food type or macronutrient composition^30^.

In addition, we let our participants choose their own speed on the treadmill, with the aim of simulating a post-prandial walk. This reflects a low-intensity activity, albeit one that makes research results transferable to real world settings. It is quite possible that a higher intensity, e.g., by means of interval training, would have led to significant improvements in ACM.

Lastly, this study is not without limitations. Firstly, ACM changes in general are known to be individually pronounced by variable sympathetic activity and different dietary strategies, as was reported in previous research^31^. Individual circadian variations in HRV abnormalities displaying DAN are known to be associated with an increased risk of ventricular arrhythmias and mortality in type 1 diabetes^25,32^. These findings emphasize the prevention of DAN by effective glycemic control^25^. Secondly, according to our previous research, we scheduled the study visits in the morning hours with the participants being overnight fasted to exclude potential interferential effects of variable sympathetic activity or impaired glycaemia^31^. In this context, a potential effect of the *Dawn phenomenon* has to be taken into consideration referring to periodic episodes of hyperglycemia and variable insulin levels occurring in the morning hours^33^. Latest research on HRV and glycemic control in T2D revealed an improved primary outcome for intensive insulin therapy in T2D with DAN highlighting the importance of optimized glycemic control in this cohort^34^. In addition, since we did not provide a standardized specification for treadmill walking exercise intensity, the exact effects of PA on ACM cannot be conclusively explained. Regarding the sample size, it must be stated that the á priori power analysis for calculated for the primary outcome of the study. Therefore, it is not guaranteed that our secondary analyses are adequately powered. Lastly, we did not collect HRV data during treadmill exercise itself, as we focused on the post-exercise effects due to variable dietary macronutrient composition.

To the best of our knowledge, this is the first study to compare the effects of a high-carbohydrate and high-fat meal with and without subsequent PA on baseline ECG and HRV parameters in people with T2D. Our results indicate that there are no clinically relevant HRV or heart rhythm abnormalities because of either meal, with PA demonstrating no relevant alteration in these parameters. Nevertheless, it should be mentioned that our study group consists of people with T2D who in general have a significantly increased risk of developing DAN during lifetime, which seems not to be adversely affected by our specific nutritional intervention during the short-term observation. These circumstances are of great public interest since specific nutrition patterns will gain great attention in the future. In this context, our observations provide valuable information for people with T2D due to specific nutrition patterns and their safe application of individual nutritional modification. The cardiac data evaluation did not reveal significant differences in ECG and HRV parameters nor enhanced cardiac stress levels demonstrating the safety of high-carbohydrate and high-fat meals with additionally optional aerobic PA.

## Conclusions

No clinically relevant changes in HRV and ECG data were found after consuming either a high-carbohydrate or high-fat meal in people with T2D. In addition, post-prandial PA also did not impair the cardiac response in this cohort. Future studies should elucidate the impact of long-term dietetic interventions with and without physical activity that might be placed before or after meal intakes, on cardiac autonomic modulation in people with T2D.

## Data Availability

Data will be provided upon reasonable request by the corresponding author.
